# Advances of Welding Technology of Glass for Electrical Applications

**DOI:** 10.3390/ma18174096

**Published:** 2025-09-01

**Authors:** Dejun Yan, Lili Ma, Jiaqi Lu, Dasen Wang, Xiaopeng Li

**Affiliations:** 1 Guangdong Provincial Key Laboratory of Advanced Welding Technology for Ships, Foshan University, Foshan 528011, China; lys@njust.edu.cn; 2 College of Materials Science and Technology, Nanjing University of Science and Technology, Nanjing 210014, China; malili0512@126.com (L.M.); 124116023919@njust.edu.cn (J.L.); 3 The Ningbo Branch of Ordnance Science Institute of China, Ningbo 315103, China; malili0512@163.com

**Keywords:** glass brazing, ultrasonic welding, CO_2_ laser, ultrashort pulse laser

## Abstract

Glass, as an amorphous material with excellent optical transparency and chemical stability, plays an irreplaceable role in modern engineering and technology fields such as semiconductor manufacturing and micro-electro-mechanical systems (MEMS). For example, borosilicate glass, with a coefficient of thermal expansion (CTE) that is close to having good thermal shock resistance and chemical stability, can be applied to MEMS packaging and aerospace fields. SiO_2_ glass exhibits excellent thermal stability, extremely low optical absorption, and high light transmittance, while also possessing strong chemical stability and extremely low dielectric loss. It is widely used in semiconductors, photolithography, and micro-optical devices. However, the stress sensitivity of traditional mechanical joints and the poor weather resistance of adhesive bonding make conventional methods unsuitable for glass joining. Welding technology, with its advantages of high joint strength, structural integrity, and scalability for mass production, has emerged as a key approach for precision glass joining. In the field of glass welding, technologies such as glass brazing, ultrasonic welding, anodic bonding, and laser welding are being widely studied and applied. With the advancement of laser technology, laser welding has emerged as a key solution to overcoming the bottlenecks of conventional processes. This paper, along with the application cases for these technologies, includes an in-depth study of common issues in glass welding, such as residual stress management and interface compatibility design, as well as prospects for the future development of glass welding technology.

## 1. Introduction

Fused silica (amorphous SiO_2_) is widely used in semiconductors, photolithography, and micro-optical devices [1] due to its excellent physicochemical properties, such as thermal stability [1], extremely low optical absorption [2], and high optical transmittance. Fused silica possesses an absorption coefficient of 90% from deep ultraviolet to infrared. It also exhibits strong chemical stability [2] and extremely low dielectric loss, making it valuable in microelectronics and communication packaging [3]. Borosilicate glass (e.g., Schott D 263^®^ M), with its ultra-low coefficient of CTE (3.25 × 10^−6^ K^−1^) [4], excellent thermal shock resistance, and superior chemical durability [5], is an ideal material for demanding industrial and scientific applications, particularly in semiconductor manufacturing, MEMS, precision optics, and aerospace [6]. Glass materials usually need to be built or bonded in numerous portions before they can be utilized in practical applications, where a single block of glass is insufficient to meet usage requirements [7].

Mechanical joining [8], adhesive bonding [9,10], glass fusion bonding [11], and thermal diffusion bonding [12] are some examples of traditional glass-joining processes. However, each of these strategies has its limitations. Mechanical joining attaches components using force or form-fit mechanisms, enabling manufacturing tolerances of less than 10 μm [13]; however, it easily leads to uneven stress on the glass, causing it to fracture. Furthermore, cracks are easily generated in working environments under alternating thermal and/or vibration loads. Adhesive bonding generally refers to the technology that achieves bonding between glasses by utilizing the curing reaction process of organic adhesives. Adhesive bonding is simple to operate and applicable to a wide range of materials. However, it has drawbacks such as being prone to bleaching and aging, and releasing harmful gases. It is difficult to use in the aerospace field and under high-temperature and high-load conditions [14].

The above-mentioned traditional joining methods are no longer capable of meeting the needs of national industrialization and the manufacture of numerous inventive devices due to a variety of technical inadequacies and limitations in processing precision. As a result, glass welding technology has become the preferred method for glass joining [9]. Currently, glass welding techniques can be divided into glass brazing [15], ultrasonic welding [16], ultrasonic-assisted welding [17], anodic bonding [18], and so on. However, these approaches are still limited by their sensitivity to process factors and material compatibility. CO_2_ lasers (10.6 μm, high absorption rate) [19] and femtosecond lasers (nonlinear absorption effects), with their high energy density, non-contact processing, and precise thermal control, have emerged as key technologies for overcoming traditional methods’ limitations [20].

This paper systematically reviews the development of glass welding technologies, analyzes the technical bottlenecks of both traditional and emerging joining methods, focuses on the mechanisms and research progress of laser welding, highlights the current challenges in glass welding, and explores future development directions in this field from the perspectives of laser–material interaction mechanisms and the development of intelligent processing systems.

## 2. A Review of Glass Welding

### 2.1. Brazing of Glass

The brazing of glass is a process method that utilizes low-melting-point filler metal to achieve bonding between glass and metal or between glass within a moderate temperature range. It is widely used in microelectronic packaging, optical devices, sensors, vacuum devices, and other fields. The basic principle is that the filler metal melts and wets the glass surface at a temperature that does not deform or soften the glass, forming a strong joint after cooling [21]. During brazing, the most important factor is the matching of the CTE between the filler metal and the base glass.

Sapphire (Al_2_O_3_), with characteristics such as high strength and high hardness, is frequently employed in spacecraft windows and micro-mechanical systems, although its application is constrained by physical dimensions. Current manufacturing technology allows for the fabrication of sapphire components up to 400 mm in size [22]; however, the material’s brittleness presents issues for machining functional components. Brazing provides a viable alternative because it allows for strong bonding between sapphire substrates at relatively low temperatures, making it easier to fabricate larger assemblies, increasing sapphire’s practical applications, and lowering fabrication costs [23].

**Figure 1:**
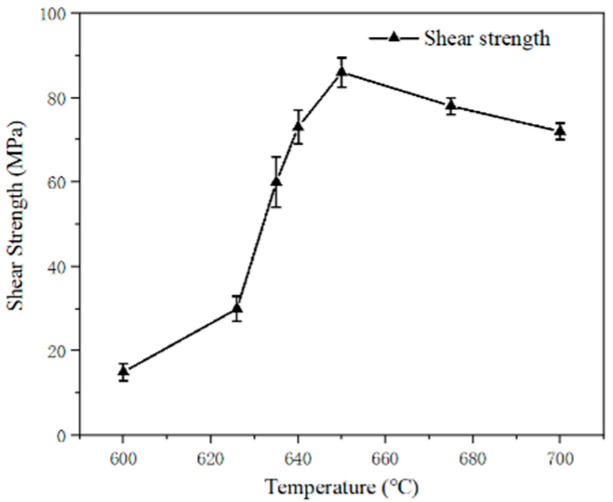
Effect of brazing temperature (t = 20 min) on the shear strength of sapphire/sapphire joint [15].

Dalton [24] reviewed the key features of glass solders suited for bonding and sealing, intending to reduce residual stresses after brazing and achieve more stable junctions. Guo [15] and colleagues successfully connected sapphire using a 50Bi_2_O_3_-40B_2_O_3_-10ZnO borate glass in the air at 600 °C to 700 °C. The results indicated that the brazing temperature has a substantial influence on the joint shear strength, as shown in Figure 1.

**Figure 2:**
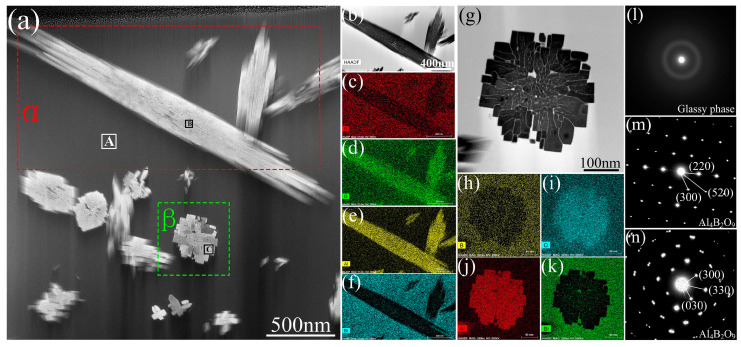
(**a**) A high-angle annular bright field (HAABF) TEM micrograph of the products within the joint bonded at 700 °C for 20 min, (**b**) and (**g**): high magnification images of specific regions, α and β, in (**a**), respectively; (**c**–**f**) and (**h**–**k**): X-ray maps of B (red), O (green), Al (yellow), and Bi (blue) at specific regions of α and β in (**a**), respectively; (**l**–**n**) selected area electron diffraction patterns (SAEDPs) of A, B, and C in (**a**), respectively [25].

As can be observed, the joining temperature has a significant influence on the shear strength. As the brazing temperature went from 600 °C to 700 °C, the joint strength initially increased and then decreased. When the brazing temperature was 600 °C, the joint shear strength was only 15 MPa. The strength continued to increase as the brazing temperature increased. When the temperature reached 635 °C (60 MPa), the shear strength increased dramatically. This could be due to the formation of finely dispersed ZnAl_2_O_4_ in the joint domain. A joint brazed at 650 °C for 20 min reaches its maximum shear strength of 85 MPa. It was observed that the shear strength of the joint reduced as the temperature increased, possibly due to the aggregation of ZnAl_2_O_4_ particles in the joint at 675 °C. Thereafter, the team [25] bonded sapphire at 700 °C in the air using 50Bi_2_O_3_-50B_2_O_3_ glass for 20 min. The joint structure is shown in Figure 2. The experimental results showed that Al_4_B_2_O_9_ is the predominant phase in the bonding zone, which is due to an interfacial interaction between the sapphire substrate and B_2_O_3_ from the glass.

**Figure 3:**
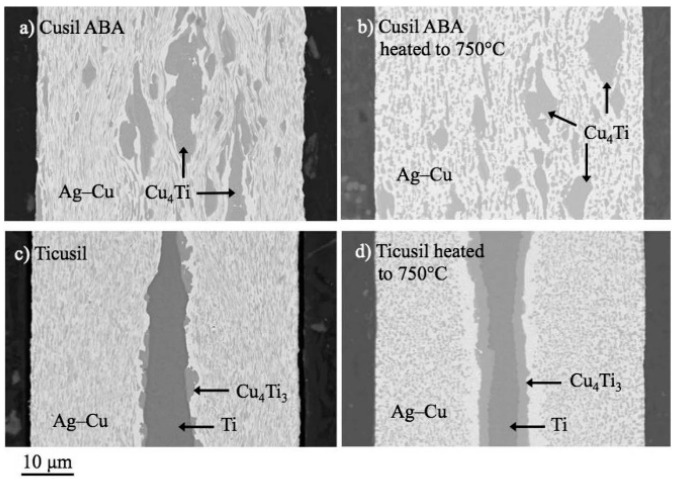
The microstructure and morphology of the solder and joint after heating to 750 °C and holding for 1 min. (**a**) Cusil active braze alloy (ABA) and (**c**) Ticusil, and (**b**,**d**) different cross-sections of the same ABAs [26].

Ali [26] studied the interactions between two commercially available Ag-Cu-Ti-based active braze alloys and sapphire substrates. In the experiment, Ag-35.3Cu-1.8Ti wt.% and Ag-26.7Cu-4.5Ti wt.% alloys were sandwiched between pieces of sapphire oriented in the R plan (1102). The assembly was heated in argon to 750~900 °C and held for 1 min. The interfacial phases of the Ag-Cu-Ti/sapphire system were then investigated using selected area electron diffraction (SAED), energy-dispersive X-ray spectroscopy (EDS), and electron energy loss spectroscopy (EELS). Research indicates that temperature variations significantly alter the Ag-Cu-Ti/sapphire interface. When the active braze alloys melt, titanium migrates to the sapphire substrate, reacting and dissolving ~33 at.% oxygen, forming a nanometer-size polycrystalline layer with a chemical composition of Ti_2_O_1−x_ (x << 1). Ti_3_Cu_3_O particles form below the Ti_2_O_1−x_ layer and develop into continuous micron-scale layers, eventually replacing it. At 845 °C, a nanometer-sized γ-TiO [27] (cubic, Fm3m, space group 225, a specific crystalline form of titanium dioxide (TiO_2_)) layer forms on the sapphire surface, establishing a characteristic Ag-Cu/Ti_3_Cu_3_O/γ-TiO/sapphire interface structure. This is similar to the interface reported in polycrystalline alumina samples bonded with active braze alloys. Figure 3 displays the cross-sectional of the active braze alloys (ABAs) before and after heating to 750 °C, demonstrating solid-state diffusion in the ABAs at this temperature.

Domestic and international research indicates that glass brazing, as a method for joining glass components, forms a stable transition layer or compound at the interface, resulting in a good joint with advantages such as high joint strength and large toughness [28]. Glass brazing operates at lower processing temperatures than standard bonding processes such as bonding/cementin, frit welding, and diffusion welding, lowering the danger of heat damage to glass substrates. However, it places relatively high demands on the brazing filler metal. If the CTE of the filler metal is mismatched, interface spalling may occur with temperature fluctuations, leading to large residual stresses in the joint. Differences in the CTE may cause interfacial delamination between the glass and the metal, especially during cooling, where the resulting residual stresses can easily lead to joint failure [1]. And, some solders may have a slight toxicity.

Furthermore, selecting the appropriate glass material is crucial, particularly for alkali-containing glasses, because different types of glass vary in their chemical composition, especially in their alkali metal content (e.g., sodium, potassium, and/or lithium) [29]. These differences result in variations in their melting points, CTE, and viscosities. These variations not only affect the flow behavior of the glass during the brazing process, but also influence its mechanical properties and thermal stability. For example, a higher alkali metal content typically lowers the melting point and increases the CTE, which can lead to stress accumulation and potential cracking during the cooling phase [29]. All of these factors significantly impact the brazing process, highlighting the importance of selecting the correct glass material to ensure optimal joint strength, durability, and thermal stability throughout the device’s service life.

### 2.2. Ultrasonic Welding

Ultrasonic waves refer to a type of extremely short mechanical wave with a wavelength of less than 2 cm in the air and a frequency greater than 20 kHz. Ultrasonic welding is a procedure that uses high-frequency mechanical vibrations to transform electrical energy into thermal energy, effectively connecting two components according to Kim [30]. During this procedure, electrical energy is converted into mechanical vibrations using a sonar electrode, which generates heat through internal and interfacial friction between the workpieces to execute welding. In ultrasonic welding, localized heating and material plasticization at the contact interface are crucial for achieving reliable bonding. Alkali-containing glass (e.g., containing Na_2_O, K_2_O, or Li_2_O) is particularly sensitive to the welding process [29,31]. The alkali metal oxide content and composition significantly affect the thermophysical and mechanical properties of the glass, including the specific heat capacity, thermal diffusivity, softening temperature, and viscoelastic behavior. For example, a higher alkali metal oxide content generally lowers the softening temperature and increases the CTE, which can promote the glass flow during welding, but also increase the risk of thermal stress and cracking upon cooling. Conversely, a lower alkali content improves the thermal stability and chemical durability, but requires higher processing temperatures. They also alter the thermal response and energy absorption characteristics of the glass during welding, thereby exerting an important influence on the joint strength and process stability [31].

Kuckert [16] successfully welded borosilicate glass and metal sheets formed of the iron-based alloy NiCo2917 using ultrasonic welding. Wangner [32] investigated borosilicate glass and titanium dioxide-coated silica glass, and for the first time, they achieved ultrasonic glass welding by the systematic optimization of welding parameters such as the welding force, welding amplitude, and welding energy. The welding procedure created roughly 1 mm^2^ weld spots that were spread across the glass’s contact surface. The maximum stress achieved in compression shear tests was 40 MPa. The researchers then employed the ultrasonic welding procedure to produce metal–glass welds with tensile shear strengths of 50 MPa.

As a pressure welding method, ultrasonic welding has the advantages of a short connection time and low cost. It is mostly suitable for polymer materials and the connection between completely different materials, especially sheet materials [33]. However, it is inferior to glass brazing in terms of its joint strength and durability. Subsequently, there have been few reports on the direct ultrasonic welding of glass.

### 2.3. Ultrasonic-Assisted Brazing

When ultrasonic waves pass through liquid solder, they can produce special sonochemical events such as ultrasonic cavitation and acoustic streaming [34]. When cavitation bubbles reach the solid surface of the base material or intermetallic compounds, they may generate localized high temperatures and pressures, supplying energy for reactions at the interface [35]. This transfers mechanical and fluid kinetic energy into thermal and chemical energy, lowering welding temperatures and reducing residual stress after welding. Ultrasonic-assisted brazing is currently widely employed in medium-to-low-temperature joining operations [36].

When ultrasonic waves pass through liquid solder, they can produce special sonochemical events such as ultrasonic cavitation and acoustic streaming [34] These ultrasonic effects mechanically disrupt oxide films and refine the molten filler microstructure, thereby improving the wettability and enhancing interfacial reactions [37,38]. The cavitation within the molten filler creates microjets and shock waves that clean the surface, enabling intimate contact between the filler and substrate [37]. Simultaneously, mechanical and fluid kinetic energy is converted into localized thermal energy, which lowers the effective brazing temperature and promotes a better filler flow and mixing. This combination not only reduces residual stress, but also leads to the formation of joints possessing high strength, uniform intermetallic compound distribution, and reliable interfacial integrity [37].

**Figure 4:**
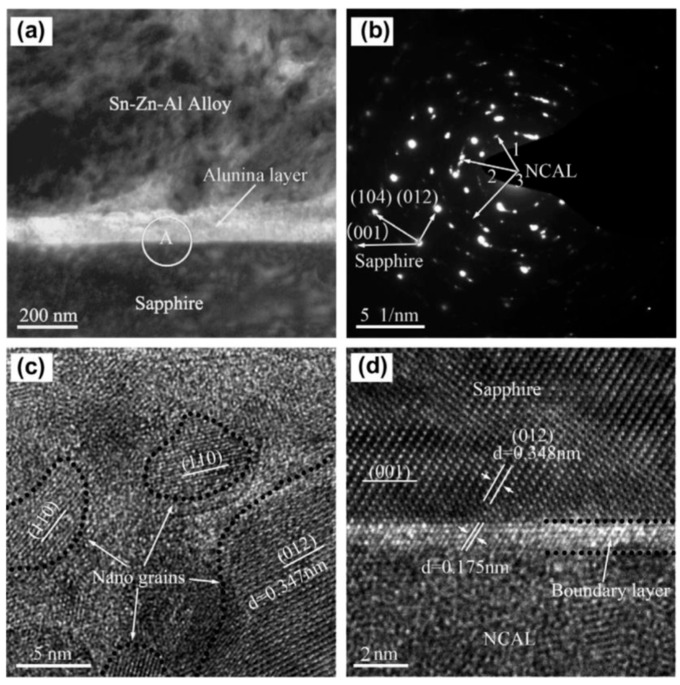
TEM image of the Sn–Zn–Al/sapphire interface [39]. (**a**) TEM image of the Sn–Zn–Al/sapphire interface that was sonicated for 1000 s. The thickness of the interfacial oxide layer was ~100 nm; (**b**) diffraction pattern of area A in (**a**). As the diaphragm of the TEM instrument has a minimum diameter of 200 nm, area A contains part of the sapphire substrate and thus the diffraction pattern includes spots corresponding to the sapphire substrate. The radii of ring 1, 2, and 3 are, respectively, 2.90, 4.34, and 4.99 nm^−1^. These correspond to interplanar spacing of 0.345, 0.230, and 0.200 nm. (**c**) HREM image of the interfacial oxide layer. The boundary of the nanocrystalline grains is profiled by dotted lines. (**d**) HREM image of the interface between the interfacial oxide layer and the sapphire substrate. A 2–3 nm thick boundary layer of the oxide layer is partially profiled by dotted lines.

Wei [39] used the ultrasonic immersion of sapphire in liquid Sn-Zn-Al to achieve sapphire welding and investigate the microstructure Sn-Zn-Al/sapphire interface. Ultrasonication accelerated the oxidation reaction of Al at the Sn-Zn-Al/sapphire interface, resulting in a nanocrystalline α-Al_2_O_3_ layer (NCAL). Near the NCAL, there is a ~2 nm boundary layer with a lattice that matches the sapphire substrate. Therefore, the NCAL allows for a seamless lattice transition from sapphire to metal. Figure 4 illustrates how the nanocrystalline α-Al_2_O_3_ grains between sapphire and the Sn-Zn-Al brazing alloy enable a seamless transition between the two materials. The joint’s shear strength can reach 43~48 MPa.

**Figure 5:**
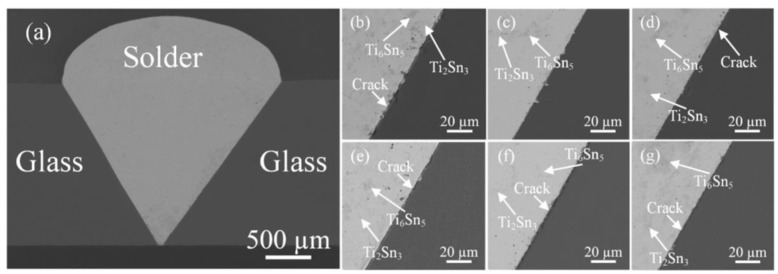
(**a**) Macroscopic morphology of SiO_2_ glass/SiO_2_ glass butt joint; microstructure of the SiO_2_ glass/Sn-2Ti brazing filler metal interface after 15 cycles of acoustic oscillation at different temperatures: (**b**) 240 °C; (**c**) 250 °C; (**d**) 270 °C; (**e**) 300 °C; (**f**) 330 °C; (**g**) 360 °C [36].

Wu [36] joined SiO_2_ glass using Sn-2Ti active filler metal via ultrasonic-assisted brazing, varying the welding temperature and the number of acoustic cycles. Figure 5a shows the macroscopic morphology of the SiO_2_/SiO_2_ butt joint, while Figure 5b–g present SEM images of the SiO_2_/Sn-2Ti interface at different temperatures with 15 acoustic cycles. Dark Ti_6_Sn_5_ and light Ti_2_Sn_3_ intermetallic compounds (IMCs) were observed near the interface. Increasing the welding temperature transformed the IMCs from strip-like clusters into coarse blocky structures, accompanied by interfacial cracks at 240 °C, 270 °C, 300 °C, 330 °C, and 360 °C. Only at 250 °C with 15 cycles did the interface appear dense, defect-free, and straight, although slight wavy grooves formed on the SiO_2_ surface due to ultrasonic cavitation.

**Figure 6:**
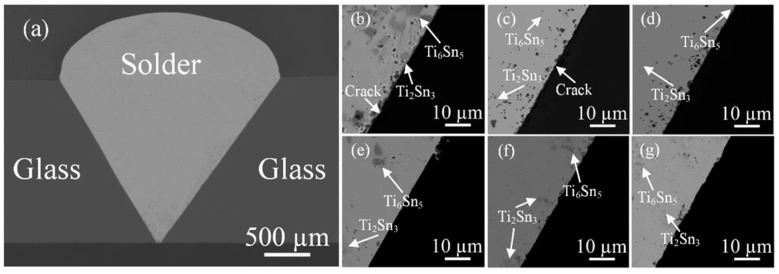
(**a**) Macromorphology of SiO_2_ glass/SiO_2_ glass butt joint; microstructure of the interface between SiO_2_ glass and Sn-2Ti filler metal at a welding temperature of 250 °C under different numbers of back-and-forth passes of the cathode: (**b**) 1 pass; (**c**) 2 passes; (**d**) 4 passes; (**e**) 5 passes; (**f**) 10 passes; and (**g**) 15 passes [36].

With the temperature fixed at 250 °C, the number of acoustic probe cycles was varied (Figure 6). At 1–2 cycles, large blocky Ti_6_Sn_5_ and Ti_2_Sn_3_ IMCs persisted near the interface and cracks were evident, preventing dense bonding. At higher cycles, the IMCs refined into uniformly distributed small particles, and the interface became dense, straight, and free of obvious cracks or pores. Nevertheless, increasing cycles led to the formation of slight surface grooves from ultrasonic action.

Ultrasonic-assisted brazing combines the advantages of ultrasonic technology and brazing technology. In this process, mechanical and fluid kinetic energy are converted into thermal energy and chemical energy, which reduces the brazing temperature, decreases post-weld residual stress, and forms high-quality joints with strength comparable to that of the base metal and good stability [36]. However, during the welding process, voids are prone to form at the solder–glass interface. Once voids are generated, the contact area will be reduced, which in turn lowers the adhesion and results in a decrease in the maximum shear strength before joint failure. In addition, prolonged ultrasonic treatment may also cause oxidation at the joint, leading to failure [40].

### 2.4. Anodic Bonding

Anodic bonding, also known as field-assisted bonding, is a solid-state bonding technique that achieves heterogeneous material joining through the synergistic effect of thermal and strong electric fields. Since Wallis first proposed this method in 1969 [41], this technique has been widely used in micro-electro-mechanical systems [42]. Anodic bonding is a superposition of physical and electrochemical processes, involving physical conditions such as strong electric fields, temperature fields, and pressure.

The anodic bonding process relies on the cleanliness and low roughness of the material surfaces. The higher the interface cleanliness, the more conducive it is to forming a high-strength bonding interface [43]. According to the different connection structures and material systems, anodic bonding is usually divided into direct anodic bonding and indirect anodic bonding. The former is commonly used for direct bonding between dissimilar materials such as glass and silicon or metals, while the latter expands its application range to glass–glass or other materials difficult to directly bond by introducing a reactive intermediate layer at the interface [44,45].

#### 2.4.1. Direct Bonding

**Figure 7:**
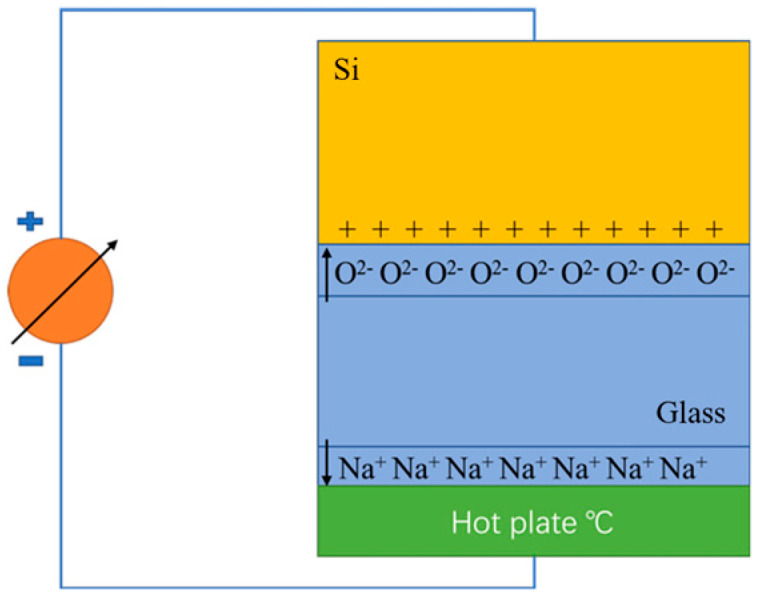
Schematic diagram of direct bonding [46].

Direct anodic bonding, also known as direct bonding or electrostatic bonding, is a solid-state joining technique that forms a permanent bond between dissimilar materials under the combined action of a high temperature and an applied electric field. The schematic diagram of the bonding process is shown in Figure 7. Mobile alkali metal ions (such as Na^+^) and O^2−^ in the glass migrate towards the electrode under the influence of the electric field, forming a depletion layer near the interface. This promotes strong electrostatic attraction and interfacial diffusion between the two materials, thereby establishing a stable bonding structure [46]. This method is particularly suitable for bonding borosilicate glass (such as Pyrex, BF33) to conductive substrates (such as silicon, Kovar alloy). Due to its ability to achieve pore-free, high-strength, and chemically stable bonding interfaces without the use of adhesives or intermediate layers, it is widely used in the fabrication of microelectromechanical systems (MEMS) packaging, hermetic packaging, and optoelectronic devices [47].

Liu [48] employed Pyrex glass and Kovar alloy (Fe-Ni-Co-based low-expansion alloys) as research subjects to investigate the process features of glass–metal direct bonding, as well as the microstructural and strength of the bonding interface under anodic bonding conditions. Pyrex glass and Kovar alloy have good connectivity under anodic bonding conditions. The Pyrex/Kovar joint fractured in the transition zone near the glass, indicating that the weak link of the joint was the interface near the glass in the transition zone. The analysis found that the connecting area between the Pyrex glass and Kovar alloy consists of glass, a transition zone, and metal. The main structure of the transition zone is FeSiO_3_ and Fe_7_SiO_10_ and its chemical composition and density significantly impact the joint strength.

**Figure 8:**
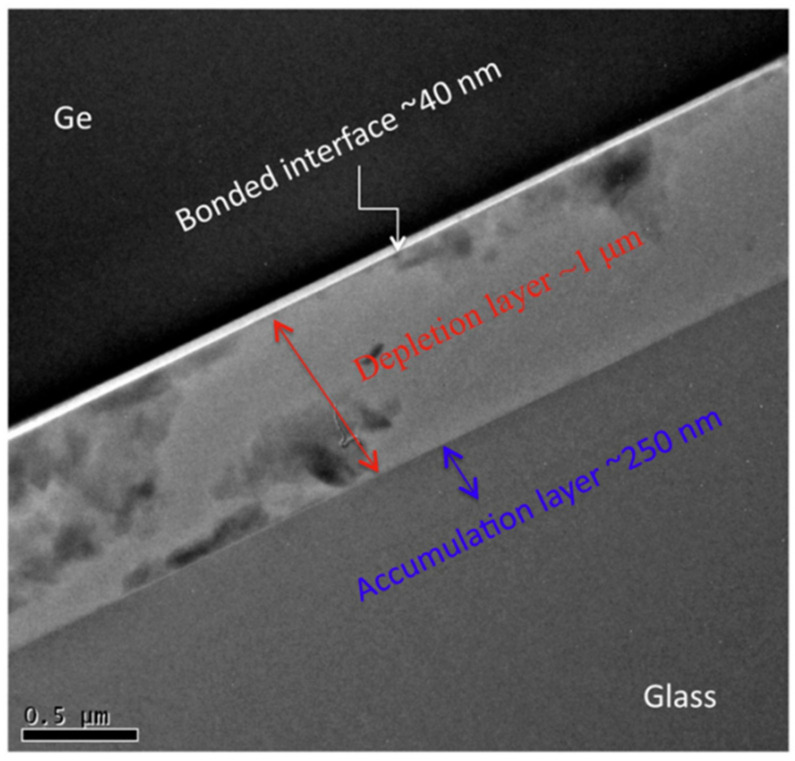
HRTEM images of bonded Ge/glass interface [50].

A glass–Kovar connection was successfully achieved by Pang [49] by direct anodic bonding studies utilizing BF33 glass and Kovar alloy. They examined the mechanical characteristics and patterns of current variations under various process conditions. According to the data, the peak current rose as the bonding temperature and voltage increased, and the joint’s bonding strength likewise increased as these factors increased. The bonding strength could be as high as 6.8 MPa at 500 °C and 1000 V. Howlader [50] successfully fabricated Ge/Pyrex glass bonded joints with a bonding strength of 9.1 MPa using hybrid plasma bonding (HPB, i.e., sequentially plasma activation followed by anodic bonding), which involves sequential plasma activation followed by anodic bonding, under a temperature of 200 °C. The HPB process consists of three steps: (1) surface activation by oxygen (O_2_)-reactive ion etching (RIE). Plasma followed by (2) surface activation by nitrogen (N_2_) microwave (MW) radicals and (3) then anodic bonding in air. At the bonding interface, a distinct three-layer structure was observed, consisting of a 1 μm thick depletion layer, a 250 nm thick alkaline layer, and a 40 nm thick germanium oxide layer, as illustrated in Figure 8. The bonding mechanism of Ge/glass is attributed to the reactions of OH molecules between the highly reactive surfaces in the sequentially plasma-activated bonding and the opposite migration of cations and anions during anodic treatment.

Tang [42] used a mixture of experimental and simulation methods to study the product at the silicon (100)/SiO_2_ anodic bonding interface, finding that amorphous SiO_2_ is formed during silicon–glass anodic bonding. Chen [51] effectively formed silicon–glass–silicon connections using a two-step anodic bonding process and examined the association between the sample tensile strength and bonding voltage. The results showed a positive correlation between the two. Furthermore, the tensile test findings showed that fractures occurred mostly on the side of the glass substrate near the second bonding interface and the strength of the second bond was lower than that of the first bond.

**Figure 9:**
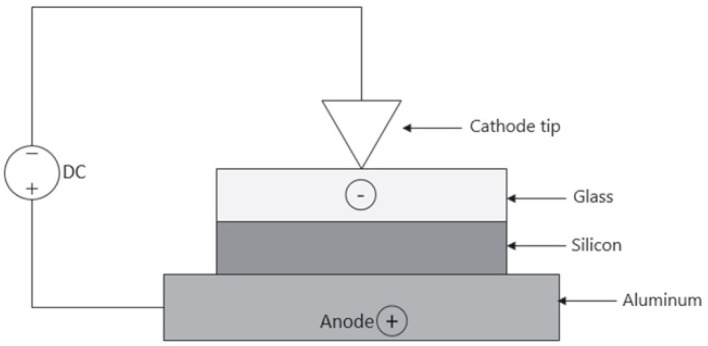
Schematic sketch of anodic bonding experimental set-up [52].

Sundaresh [52] developed dependable experimental equipment that successfully bonded silicon and Pyrex glass by direct anodic bonding under air circumstances. For anodic bonding, the Si side is made positive with respect to the glass, as shown in Figure 9. The micromanipulator tip is brought into contact with the glass substrate at the center to provide the field necessary for bonding. If the applied voltage is large enough to activate substantial Na^+^ drift, the magnitude of the voltage needed to obtain bonding will become independent of the glass thickness. The electrostatic forces pull the surfaces into close contact, allowing the formation of atomic bonds. The author achieved a tensile strength of 1134 × 10^4^ Nm^−2^ for the joint at 420 °C and 800 V.

Since the quality of the wafer surface directly affects the bonding process, and any impurity particles or oxide layers on the wafer surface will decrease the bonding efficiency and strength, the surface cleaning process can remove these impurity particles and oxide layers to obtain interfacial properties suitable for bonding. Du [47] used three solutions commonly used for cleaning—degreasing solution (Trichloroethylene), piranha solution (hydrogen peroxide solution and concentrated sulfuric acid at a ratio of 6:1), and RCA solution (hydrogen peroxide solution and the ammonium hydroxide solution were mixed in a ratio of 1:1.2)—to treat the wafer surface and perform direct anodic bonding with Pyrex glass. The results showed that the surface of the material cleaned with RCA solution would form more dangling bonds and the roughness would also be reduced, achieving higher bonding strength.

Jia [53] successfully achieved a reliable connection between aluminum foil and glass with a thickness ranging from 15 to 100 μm through the direct anodic bonding method under the bonding parameters of a bonding temperature of 450 °C, with a voltage of 1000 V, and a bonding time of 1 min. The maximum tensile strength occurred when the thickness of the aluminum foil was 30 μm. It was 12.6 MPa and the joint fracture always occurred inside the glass matrix.

#### 2.4.2. Indirect Bonding

However, anodic bonding between materials with significant differences in their thermophysical properties or weak interfacial reactivity is generally difficult to achieve. This is because residual stress tends to be large, the interfacial ion migration capability is insufficient, and an effective electric field is difficult to form at the interface [54]. To address this issue, an intermediate layer can be introduced at the interface for bonding. This method, known as indirect bonding, enables the connection of materials that are otherwise difficult to bond, expands the selection range of material combinations, and improves the bonding reliability and hermeticity [45].

Unno [55] utilizes Al film as an intermediary layer, where the diamond film may be anodically bonded to Pyrex glass at a bonding temperature of 500 °C and an electric voltage of 600 V.

**Figure 10:**
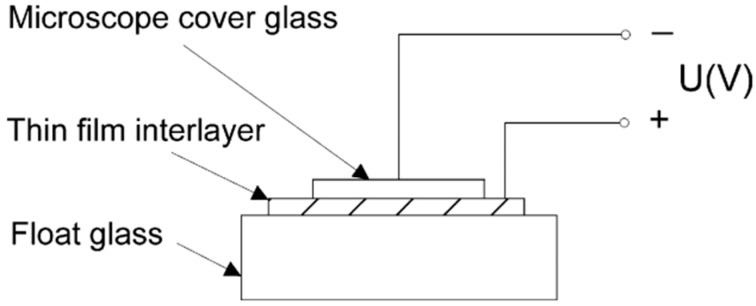
Schematic diagram of anodic bonding of glass plates using a thin-film interlayer [57].

Mrozek [56] employed vacuum-deposited titanium thin films for the indirect anodic bonding of glass plates with an interlayer in all-transparent devices. The author deposited Ti films with thicknesses of 40 nm and 80 nm onto the top surface of one of the joined glass slabs under vacuum conditions of 10^−4^ hPa and 10^−5^ hPa, respectively, prior to bonding. The glass plates were then anodically bonded using the Ti thin film interlayer, as shown in Figure 10, where the Ti thin film is the anode and the microscope cover glass is the cathode. The microscope top cover glass was anodically bonded to the float glass using a thin-film interlayer. A seal with a tensile strength of 25 MPa was fabricated finally. After that, Mrozek [57] employed magnetron sputtering TiNx thin films as an interlayer and accomplished the anodic bonding of glass plates at 430 °C and 50 V, resulting in glass–glass junctions with a tensile strength of 23.5 MPa. Furthermore, the study discovered that a thin film of a 22 nm thickness is used to obtain the optimal optical properties of the bond in this scenario.

**Figure 11:**
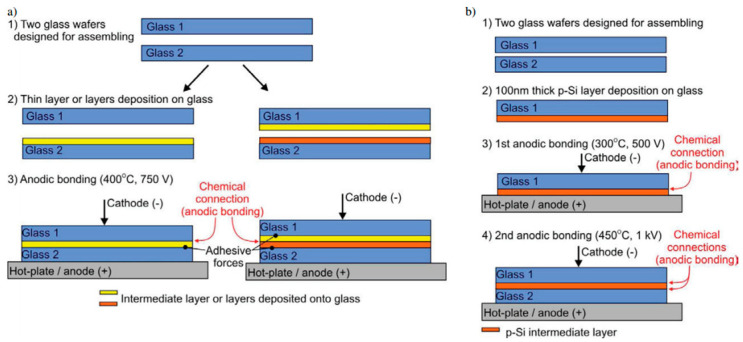
Flow chart for glass-to-glass assembly: (**a**) 1. Prepare two glass wafers. 2. Deposition of thin film layer/layers onto glass wafer/wafers. 3. Glass-to-glass anodic bonding. (**b**) Newly invented procedure: 1. Preparation of two glass wafers. 2. Deposition of a thin p-Si layer on one glass wafer. 3. Thermal treatment of the p-Si layer (anodic bonding). 4. Glass-to-glass anodic bonding [58].

Berthold [54] employed many thin films as an intermediary layer for binding glass to glass. The intermediate layers chosen include amorphous silicon, polycrystalline silicon, silicon nitride, silicon carbide, and silicon dioxide, as well as combinations of two or three of the elements listed above. However, at least one of the connections between the intermediate layer and the glass is made just through adhesive force. Knapkiewicz [58] expanded on this bonding process and suggested a methodology for glass-to-glass integration, employing indirect anodic bonding through a thin polysilicon layer (p-Si) as the intermediate layer. Figure 11 depicts the connection diagram. The approach is distinguished by high-quality bonding generated by a unique p-Si forming procedure. Breaking tests on test samples revealed a bonding force greater than 40 MPa.

Lin [59] created a silicon–glass–silicon three-layer anodic bonding structure with a Si_3_N_4_ film. The bonding area exceeded 90% under a 400 °C bonding temperature, 1200 V voltage, and 450 MPa pressure. This study improved the anodic bonding quality in structures containing Si_3_N_4_ films, addressing challenges of a limited bonding area and low bonding strength.

Tang [60] deposited 1 μm thick, low-stress (<10 MPa) amorphous SiC on the silicon substrate using PECVD. The silicon substrate was coated with a Guo Å thick conductive layer of W as the upper electrode and a 5000 Å thick dielectric layer of SiO_2_. Bonding with glass was successful at 400 °C, a 1000 N pressure, and 1300 V voltage. Building on this, the group used plasma-enhanced chemical-vapor-deposited silicon carbide (PE-SiC) as the intermediate layer to further achieve the traditional silicon–glass indirect anodic bonding and examined how the thickness of the intermediate layer affected the bonding performance. The findings demonstrated that the bonding strength dropped with the increasing PE-SiC thickness. Subsequent tests showed that this technique reduced the stress gradient by 24.6 MPa µm^−1^ and raised the interlayer’s tensile stress by 70.7 MPa in comparison to traditional Si/glass anodic bonding.

Elefaey [61] employed a layer of liquid tin-based solder between the glass and metal component of the junction to produce low-stress bonding between titanium alloy and 3 mm thick soda–lime float glass. The titanium alloy and glass are deposited with a nickel coating to increase the contact between the surfaces. The study found that Ni_3_Sn_4_ intermetallic compounds were formed at the interface, significantly lowering internal stress at the interface, and the shear strength of the joint reached 21 MPa.

**Figure 12:**
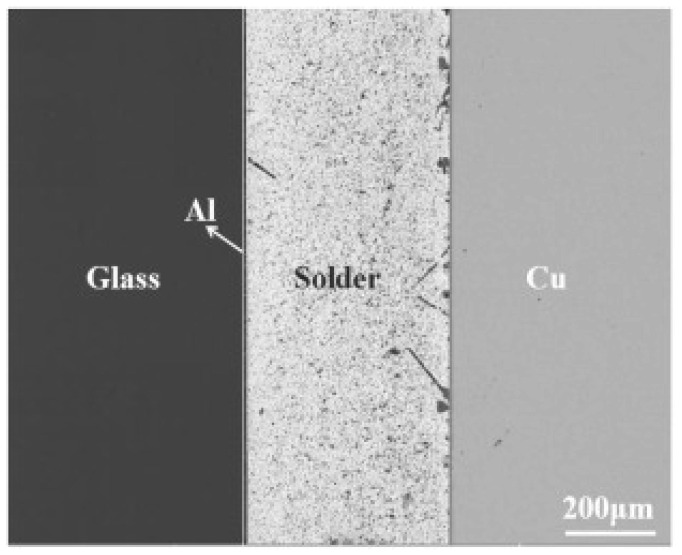
SEM image of the glass/Sn-9Zn/Cu joint at 240 °C for 5 min [62].

Hu [62] proposed a new coupling method for glass–metal joining. First, the glass and Al were effectively linked using an anodic bonding technique and then, the glass and Cu were bonded together using a eutectic Sn-9Zn solder, achieving indirect bonding between glass and copper. The bonding joint is shown in Figure 12. At 240 °C for 5 min, the joint reached its maximum strength of 12.7 MPa.

Anodic bonding is widely used in micro-electro-mechanical systems (MEMS) [63,64], micro-mechanical pressure sensors, and atomic gyroscopes [65] due to its high quality [66], simple operation, and low bonding temperature [67]. However, it is highly sensitive to the CTE values of the bonded components. Furthermore, it requires high smoothness and flatness of the bonding material surfaces, which impose certain limitations in industrial production. The careful adjustment of process parameters such as the voltage and temperature is required to achieve the actual bonding requirements [68].

Although methods such as glass brazing, ultrasonic welding, and anodic bonding have achieved effective glass joining in different scenarios, they still exhibit bottlenecks. Brazing requires the precise control of temperature and solder composition, and residual stresses easily arise due to mismatches in thermal expansion [28]. Ultrasonic welding imposes strict requirements on the surface quality of materials [69]. Anodic bonding relies on the synergistic effect of electric fields and temperature, making it difficult to join the same type of glass or materials with large differences in thermal properties [70]. These methods are either limited by the process complexity or material adaptability, failing to meet the high-strength, high-precision, and multifunctional requirements of modern precision manufacturing for glass joining.

With the development and advancement of optical technology, laser welding technology has gradually become the key to breaking through the bottlenecks of traditional joining techniques, owing to its unique characteristics such as non-contact processing, high energy density, and precise thermal control. Laser welding utilizes coherent laser beams to achieve localized rapid heating, avoiding extensive thermal damage caused by traditional methods [71]. Meanwhile, it enables the precise control of the welding process by adjusting laser parameters, making it particularly suitable for joining glass in precision devices such as optical components and micro-electro-mechanical systems (MEMS). From CO_2_ laser welding [72] and nanosecond (NS) lasers [73] to nonlinear absorption welding with ultrashort pulse (USP) lasers [74], such as femtosecond lasers, it is driving the development of glass welding towards low thermal stress, high transparency, and cross-material compatibility. The following sections systematically elaborate on the technical principles, research progress, and application prospects of laser welding in glass joining from the dimensions of a CO_2_ laser and USP laser.

### 2.5. Application of Lasers in Glass Welding

Laser welding has steadily developed as a major topic of research in China and abroad as optical technology has advanced [75]. Laser radiation’s coherence and monochromaticity [76], combined with its higher power levels than most other light sources, give it a unique instrument for precision thermal processing. Using advanced laser technology for material processing can enhance the quality of workpieces to levels unreachable by other methods. Laser welding uses a high-energy-density laser beam to heat the materials being connected. Its high power density allows it to reduce the heated area while keeping the geometric shape of the workpiece [77].

Since the introduction of lasers in the 1960s, the number of laser types has rapidly expanded, as has the spectrum of materials that can be processed. Laser welding, as one of the principal uses of laser technology, did not become widely used in precision manufacturing industries such as electrical and electronic engineering and instrumentation until sophisticated laser technologies such as CO_2_ lasers and fiber lasers matured [78].

#### 2.5.1. Glass–Glass Welding by NS Laser

NS lasers have a smaller heat-affected zone than CO_2_ laser welding due to their wavelength falling within the glass transmission window. However, it is often required to insert an absorbent layer within the gap between the materials to be welded. The usual form of ns laser welding is the NS Nd: YAG long-pulse laser [79].

Sun [73] used ANSYS simulation software (https://www.ansys.com/) to model the Nd: YAG laser welding process of soda–lime glass plates and successfully welded flat vacuum glass using an Nd: YAG laser. Following Sun Gan’s work, He [80] investigated the effects of the pulse current, pulse width, pulse frequency, and welding speed on the molten layer of vacuum flat glass, the interfacial reaction wetting layer, and the mechanical properties. The study made use of low-melting-point glass solder based on the PbO-TiO_2_-SiO_2_-R_X_O_y_ combination, as well as regular soda–lime glass. The findings revealed that when the pulse current was 16 A, the pulse width was 2 ms, the pulse frequency was 18 Hz, and the welding speed was 900 mm/min, defects in welding were minor and the sealing quality was outstanding.

**Figure 13:**
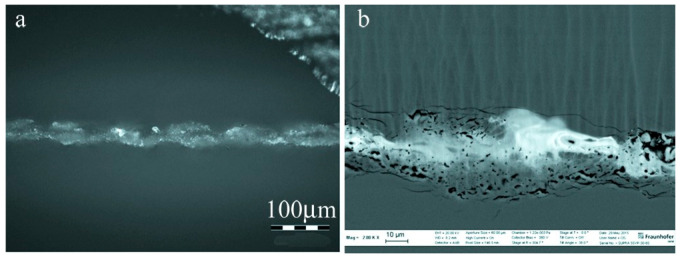
Cross-sectional micrograph of a soldered sample with BTS binder applied to the bottom substrate. (**a**) Optical micrograph and (**b**) SEM micrograph employing an annular scintillator detector [81].

De Pablos-Martin [81] employed 1064 nm and 532 nm 5 ns Nd: YAG laser welding of two samples of 1 mm thick borosilicate glass through irradiation with an NS pulsed laser to evaluate the influence of the presence or absence of a Ti absorption layer at the interface between the glass plates. The results revealed that without the Ti absorption layer, light scattering at the interface resulted in micro-cracks and micro-holes. The employment of a Ti thin layer as an absorber increases the quality of the interface. In addition, the team [79] also investigated the use of fresnoite glass films (2BaO-TiO_2_-2SiO_2_, BTS) as absorbers, employing a 1064 nm 5 ns Nd: YAG laser to weld two 500 nm thick fused silica glass pieces. The analysis revealed that with the addition of the additive, no significant cracks or voids were observed at the joint, as shown in Figure 13. The residual tensile stress at the joint is reduced by 85% compared to direct welding, dropping from 47 MPa to 7 MPa. However, compared with the fused quartz substrate, the transmittance of the welded substrate decreases by 2% to 10%.

Zhang [82] used a 1064 nm NS laser to weld two 1 mm thick soda–lime glass substrates, with a 14 nm thick titanium thin-film coating applied between them to assist the welding process. Scanning electron microscopy (SEM) revealed no cracks or damage at the joint. Through the laser process, the welded area is not only highly transparent, but does not significantly modify the transmittance-varying trend over the test wavelength range, except in that the transmittance is slightly reduced. The maximum change rate of transmittance of the welded zone is 8.88% in the wavelength range of 400~1800 nm.

#### 2.5.2. Glass–Glass Welding by CO_2_ Laser

CO_2_ laser welding utilizes a 10.6 μm mid-infrared laser that is strongly absorbed by glass [83], enabling efficient localized heating and material melting at the surface [84]. In this process, laser energy is converted to heat at the glass interface, causing softening and melting, which allows for direct bonding or hermetic sealing without intermediate layers. This non-contact and high-efficiency approach is promising for glass joining, especially applicable to various transparent substrates and packaging processes [72].

Beyond these basic principles, the practical implementation of CO_2_ laser welding requires the careful tailoring of process parameters and thermal management to balance joint formation, optical quality, and mechanical reliability. While CO_2_ welding excels at the high-throughput formation of macroscopic seams and hermetic seals for displays, substrates, and packaging, its wider thermal footprint compared with ultrashort-pulse methods necessitates post-process strategies (annealing and surface finishing) and rigorous reliability testing to ensure its long-term optical and mechanical performance.

In their investigation, Nam [85] used a CO_2_ laser to cut and seal quartz and borosilicate capillary glass tubes with a diameter of 1.4 mm and a thickness of 0.25 mm. The experimental results revealed that: (1) The laser power, laser spot size, exposure time, rotation speed of the glass tube, and exposure time all have a significant effect on the quality of glass melting and sealing. (2) The laser power required for sealing the quartz tube is approximately three times that of the borosilicate tube. However, the borosilicate tube has a slightly higher cutting power than the quartz tube.

Tang [86] used a CO_2_ laser as a heat source to seal the medicinal ampoules. Using ANSYS software, they ran numerical simulations of the temperature and stress fields during the laser thermal sealing process, obtained temperature field and thermal stress distributions, and successfully sealed the ampoules with a CO_2_ laser and a thermal drawing sealing method.

Levesque [87] used a CO_2_ laser to spot-weld glass sheets in one or more locations. This approach allows for extremely precise angle welding while still providing outstanding stability. The results reveal that the joint strength ranges from 10 kg to 30 kg per spot. An interferometer was employed to measure the surface flatness before and after welding, revealing that the surface undulation deviation of the glass remained within λ/10 of the interferometer’s laser wavelength, approximately 63.28 nm. It is evident that the welding process has a minimal impact on the glass surface quality.

Wang [88] used a low-power 40 kHz CO_2_ pulsed laser to heat ultra-thin fused silica glass that was 25 μm thick to the end face of fused silica capillaries. The experiment looked at how the welding process was affected by laser power, preheating, and annealing. The findings demonstrated that while too low laser power would result in insufficient heat absorption by the fused silica glass to reach its softening point, preventing it from reaching a molten state and making thermal fusion sealing impossible, too high laser power would cause the ultra-thin fused silica glass to vaporize directly, leading to welding failure. Furthermore, positive-focus preheating substantially reduced issues like the cracking and fracturing of the ultra-thin fused silica glass during welding. Negative-focus annealing efficiently reduced residual stress, minimizing the influence of residual thermal stress on the fused silica glass performance during welding.

Cai [89] used the ANSYS program to simulate the temperature field distribution during the CO_2_ laser welding of quartz glass plates. They conducted simulation studies to assess the feasibility of laser welding ultra-thin fused quartz glass, offering theoretical guidelines for the employment of laser technology in this procedure.

**Figure 14:**
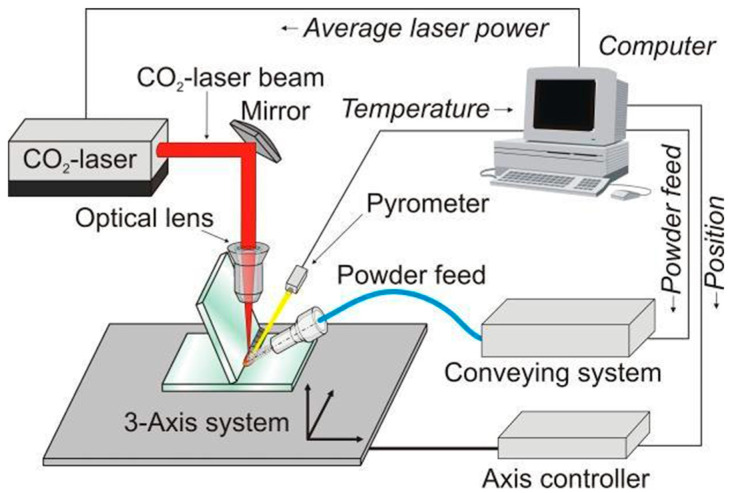
Schematic of the experimental setup [90] (reproduced from [90] with the permission of the Laser Institute of America).

**Figure 15:**
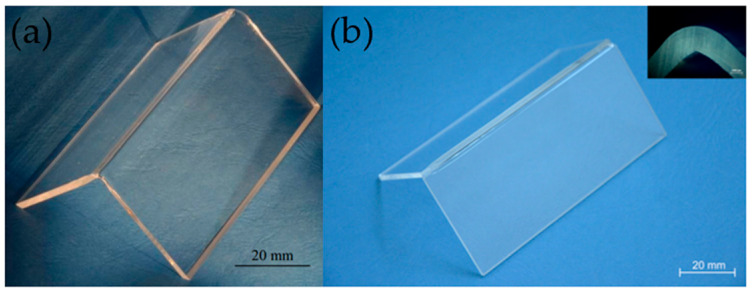
(**a**) Quartz glass corner joints welded with powder additives under controlled temperature [90] (reproduced from [90] with the permission of the Laser Institute of America); (**b**) quartz glass corner joints welded with glass fiber [91].

The Pohl team [90] investigated a temperature-controlled quartz glass welding process with a powder additive. Figure 14 displays a schematic of the experimental setup, whereas Figure 15a depicts the welded components. During the welding operation, the control system adjusts the temperature in the welding zone to near the softening point. To avoid a glass-like fracture, the joint between the two glass pieces was warmed before welding, and the sample was annealed afterward. The findings revealed that the size of the powder additive particles has a significant impact on the welding process’ stability and the powder utility rate. The best welding outcomes were attained with powder particle sizes of 250 µm. In subsequent investigations, the team employed glass fibers to fill the gap between two fused quartz glass pieces [91] and a CO_2_ laser was used to melt glass fibers and partially melt glass plates, resulting in successful seamless welding; Figure 15 depicts the welded components (Figure 15b). Real-time temperature measurements throughout the experiment revealed that preheating the glass plates to 1220 °C before welding successfully reduced cracking during the welding operation.

Zhang [92] used CO_2_ laser welding to connect the optical fiber to the collimator tube in high-temperature F-P fiber-optic pressure sensors. First, the cut optical fiber was placed into the collimator tube until its end face came into touch with the sensing structure’s bottom surface. Then, laser pulses with a specific power were used to pre-fix and execute 360° hermetic welding between the fiber and the collimator tube. The results showed that the CO_2_ laser welding process allowed for the reliable packaging of high-temperature F-P fiber-optic pressure sensors, which maintained a stable performance at temperatures as high as 300 °C.

Sleiman [72] performed angle and butt welds of flat borosilicate glass substrates using CO_2_ laser radiation by heating the welding area and inserting a borosilicate rod into the weld zone at a precise angle. The most suitable parameters, resulting in both a low contact angle of <30° and the lowest possible process temperatures of <1500 °C, are obtained at rod feeding rates of 150~200 mm/min.

In conclusion, prior research has not only encompassed various techniques and procedures in the field of CO_2_ laser welding, but has also looked at temperature-controlled welding and the usage of bridge materials. These investigations looked into the effect of various process factors on the welding quality and suggested answers to problems encountered during the CO_2_ laser welding process, ultimately furthering the development and application of CO_2_ laser glass welding technology.

#### 2.5.3. Glass–Glass Welding by USP Laser

USP lasers have pulse lengths ranging from picoseconds (10^−12^ s) to femtoseconds (10^−15^ s). Due to their extraordinarily high peak power density, they are increasingly being used in glass welding applications. USP glass welding is a technique for creating high-strength, hermetically sealed joints. By focusing the USP laser near the interface of two glass pieces, the high-energy laser at the focal point causes the multiphoton ionization of the glass material. Ionized free electrons absorb photon energy from the laser and accelerate, colliding with other atoms and causing avalanche ionization. The resulting ions and the non-ionized regions experience a temperature rise through electron–phonon coupling, reaching the material’s melting point and thereby enabling welding [93]. By this nonlinear absorption effect that occurs under extreme conditions [94,95], the glass joint is formed.

Femtosecond laser welding employs ultrashort pulses (typically tens to hundreds of femtoseconds) that generate extremely high peak powers, enabling localized energy deposition within transparent glass via multiphoton absorption and photoionization. At the focal volume, transient plasma formation and nonlinear absorption occur, confining energy to a micrometer region and triggering ultrafast electron–phonon coupling or localized melting with negligible thermal diffusion. The outcome is an extremely narrow heat-affected zone, minimal material migration, and high geometric precision—well suited for micro interconnects, photonic components, and interfaces demanding high optical clarity.

**Figure 16:**
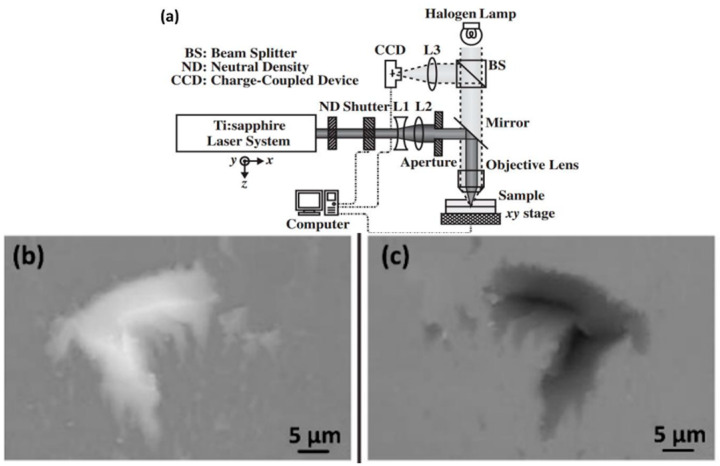
(**a**) Schematic diagram of the femtosecond laser micro-welding system; samples separated by shear external force; (**b**) lower surface of the upper sample; and (**c**) upper surface of the lower sample [96].

Tamaki [96] was the first to describe USP glass welding. Figure 16 shows a schematic of the study team’s processing system. The laser system included a Ti: sapphire oscillator and a regenerative amplifier. Tamaki used welding conditions with a feed rate of 5 μm/s, repetition rate of 1 kHz, pulse duration of 100 fs, and wavelength of 800 nm. The sequence of femtosecond laser pulses was directly focused onto the interface of two optically contacted transparent quartz glass pieces using a 10× microscope objective, inducing the nonlinear absorption of the material in the focal region and creating a strong bond between the samples at the focal point. The geometry of the weld line and welding speed were precisely controlled using a computer-controlled displacement stage. For the first time, the study team successfully welded transparent quartz glass without incorporating a light-absorbing layer.

Early demonstrations by Tamaki et al. showed that femtosecond pulses could directly weld transparent quartz glass, proving the feasibility of ultra-short-pulse bonding, but at the cost of a very low processing speed and limited joint dimensions.

**Figure 17:**
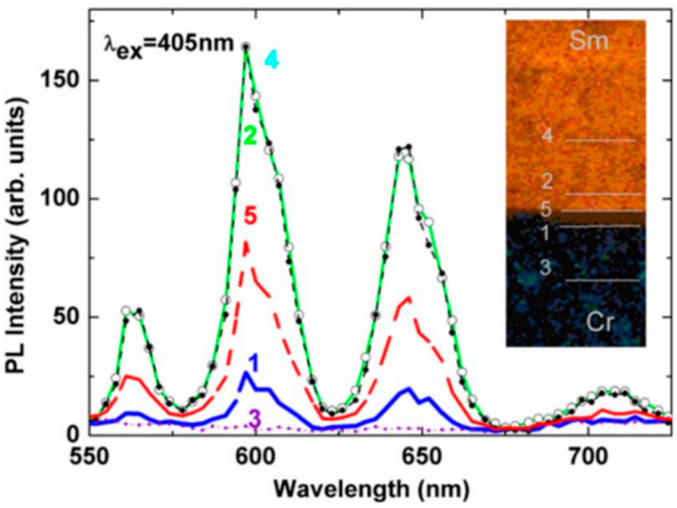
Fluorescence spectra near the interface of Sm: sodium borate and Cr-doped sodium borate near the interface: 405 nm excitation (1: 20 µm below; 2: 20 µm above; 3: 100 µm below; 4: 100 µm above; and 5: interface in the insert) [98].

Subsequently, there has been an increase in research into glass welding using USP Lasers. Watanabe [97] employed a femtosecond laser to achieve direct welding between two different transparent glass materials, quartz glass and borosilicate glass. Younshil found that after femtosecond laser treatment, ion migration occurred at the interface between the two glass components in the welding of rare earth-doped borosilicate glass [98], as illustrated in Figure 17. Watanabe and co-workers expanded the applicability to dissimilar glass systems, highlighting the flexibility of femtosecond welding, but also revealing sensitivity to mismatch in thermal and optical properties.

Itoh [99] employed femtosecond laser welding technology to weld a borosilicate glass piece with dimensions of 5 mm × 2 mm × 0.5 mm onto alumina ceramics. The welded glass–ceramic packaging sample was subjected to a helium leak test, confirming that the sample’s hermeticity was <1.0 × 10^−9^ Pa. In addition, 100 thermal cycle tests were conducted, each lasting 30 min with a temperature range of 50 °C to 125 °C. The results showed that the airtightness of the sealed cavity formed by welding did not decrease significantly. More refined investigations by Itoh et al. indicated that crack formation and bubble entrapment were strongly governed by the pulse energy and focusing conditions, pointing to the need for the precise control of nonlinear absorption and thermal accumulation.

**Figure 18:**
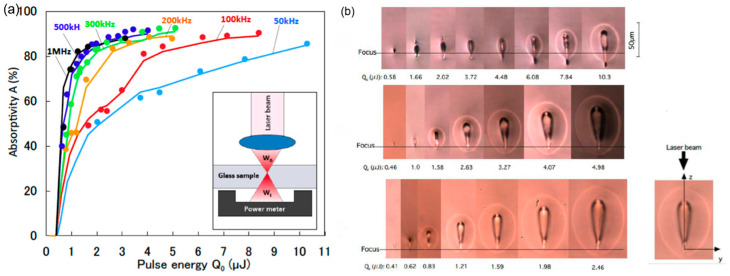
(**a**) Nonlinear absorptivity vs. pulse energy at different pulse repetition rates. (**b**) Morphology of the laser-welded area vs. pulse energy at different pulse repetition rates [101].

The absorption properties of transparent materials are regarded as a critical aspect determining the quality of USP laser micro-welding. As a result, studying these features under various laser conditions is very relevant [100]. Miyamoto [101] explored how laser factors such as pulse energy and pulse repetition rates affect the welding performance during the femtosecond pulse laser welding of borosilicate glass. The experimental findings are displayed in Figure 18. The results reveal that as the laser’s single-pulse energy and frequency increase, so does the nonlinear absorptivity of the glass material and the heat accumulation effect (Figure 18a). Specifically, this is reflected in a larger molten zone during laser welding, as well as an extended laser-induced heat-impacted zone (Figure 18b).

**Figure 19:**
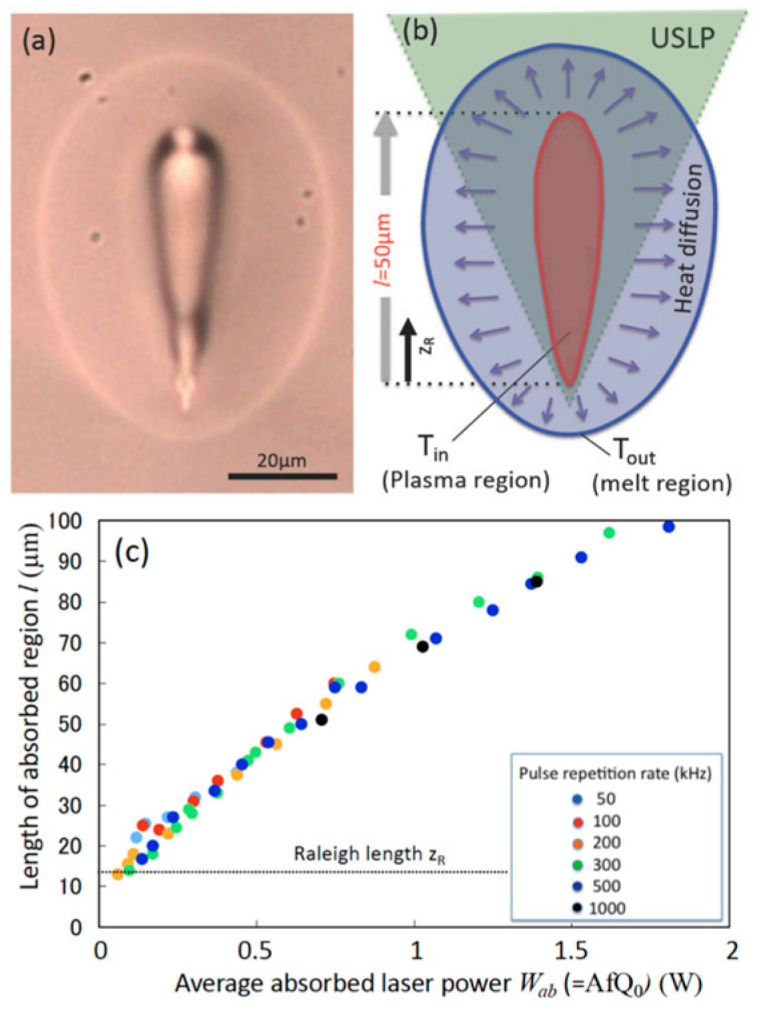
(**a**) Cross-section of weld region by USP laser showing dual structure. (**b**) Simulated contours of inner and outer structures corresponding to the laser absorption and the melted region, respectively (Z_R_ = Rayleigh length). (**c**) Length of inner structure l plotted vs. absorbed laser power Wab. As W_ab_ increases, l increases where the absorbing plasma moves dynamically (f = 500 kHz, τp = 10 ps, λ = 1064 nm, Q_0_ = 1.59 µJ, v = 20 mm/s; D263) [102].

Subsequently, the team qualitatively analyzed the crack-free conditions in glass USP welding during heating and cooling processes based on a thermal stress model [102]. The results show that when a dual structure is formed at the welding location, cracks can be prevented more safely. As shown in Figure 19, the dual structure consists of an elliptical outer structure (molten region) and a teardrop-shaped inner structure (plasma region) (Figure 19a). The laser energy is absorbed in the inner structure (plasma region), where the network modifier is enriched around its contour since the diffusion coefficient of the network modifiers is larger than that of the network formers. It can be seen from Figure 19b that the length l of the laser absorption zone is significantly longer than the Rayleigh length Z_R_ and increases with the increase in the absorbed laser power W_ab_ (Figure 19c).

Miyamoto demonstrated that increasing the single-pulse energy and repetition rate enhances nonlinear absorptivity and heat accumulation, thereby enlarging the molten region and extending the heat-affected zone. While this facilitates stronger bonding, it also raises risks of microcracking and optical distortion.

Richter [103] used a femtosecond laser with a 515 nm wavelength, 450 fs pulse width, and 9.4 MHz repetition rate to weld quartz glass. Ultrashort laser pulses with high repetition rates may melt glass and generate strong bonds. Such strong bonding allows for localized and application-specific glass joining. They also stated that in continuous welding, cracks caused by thermal stress propagate along the continuous weld seam, but the margins of weld zones created by spot welding effectively limit the propagation of stress-induced cracks. Cvevek [104] used a picosecond laser to weld two 1 mm thick borosilicate glass plates and studied the impact of the defocus distance on the welding strength. They determined that the highest allowed defocus distance is 20 μm. In the same year, Vallée [9] successfully welded fused silica and BK7 using an ultrafast laser with a wavelength of 790 nm, a pulse width of 55 fs, and a repetition rate of 250 kHz, achieving weld seams with good stability. The Richter team achieved higher-quality welds and improved mechanical integrity by optimizing the repetition rate and scanning strategy, demonstrating that by optimizing and controlling the distribution of thermal stress, crack risks can be effectively reduced and superior joints can be obtained.

Femtosecond laser welding has attracted extensive attention due to its ability to confine energy deposition within micrometer-scale volumes through multiphoton absorption and nonlinear ionization, minimizing thermal diffusion and enabling highly precise seams. The above studies indicate that femtosecond welding offers unique advantages in spatial energy confinement and seam precision, yet it remains susceptible to stress-induced defects.

Picosecond laser welding, occupying the temporal regime between femtosecond and nanosecond pulses, offers a compromise between the precision and throughput. Early studies showed that picosecond pulses can join thicker glass substrates with higher energy utilization and more stable pulse conditions.

**Figure 20:**
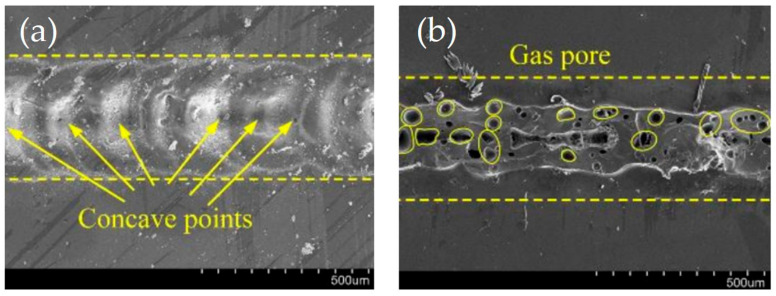
Brittle fracture (**a**). Ductile fracture (**b**) [105].

Recent investigations have integrated experimental, modeling, and industrial perspectives to advance the understanding of ultrafast laser welding. Shu [105] investigated the welding of borosilicate glass using a picosecond laser. By analyzing the relationship between the weld seam’s microstructure (as shown in Figure 20) and the fracture mode, a fracture model based on the microstructure was established. It was found that insufficient laser power and an excessive welding speed lead to insufficient energy input, which easily results in a brittle fracture. At this point, the weld zone exhibits an ordered concave structure, accompanied by particulates formed by gasified material cooling, with no obvious adhesions. When the energy is sufficient, the glass melts completely, the elements diffuse uniformly, and ductile fracture characteristics are formed. A large amount of adhesion which peeled off due to the shear test is present at the weld, with a small number of pores and micro-voids. It is concluded that in USP laser welding, the fracture mode is determined by the microstructure, and both the energy input and heat accumulation affect its microstructure. Shu’s work demonstrated that the weld microstructure is directly related to the energy input during the welding process, and a sufficient energy input facilitates the formation of a uniformly diffused weld microstructure.

Tan [106] employed a transient thermal model and a fixed support model, using the ANSYS Workbench (finite element analysis software) to simulate the temperature field, residual stress, and strain field of quartz glass directly joined by a femtosecond laser with a Gaussian heat source model. Through the analysis, the maximum stress input by the laser was only 16.474 KPa, which did not reach the fracture threshold of quartz glass. The influence of a magnetic field on the welding effect was also simulated, and it was found that the thermal stress and residual stress significantly decreased after applying a magnetic field. Tan’s finite element simulations indicate that auxiliary measures can further influence the formation of the weld microstructure.

Kaiser E [107] focused on femtosecond laser glass welding technology, pointing out that this technology has met the conditions for industrial application, and noted that TRUMPF Laser GmbH adopted the ultrafast laser welding process to weld the assembled silica glass protective covers. The tested fracture strength of the resulting protective covers exceeds twice the strength required for the tested components. Moreover, this process has been industrialized since 2016, enabling the independent production of laser cables. The thickness of the glass covers can reach 6 mm, which can transmit an average laser power of up to 6 kW.

**Figure 21:**
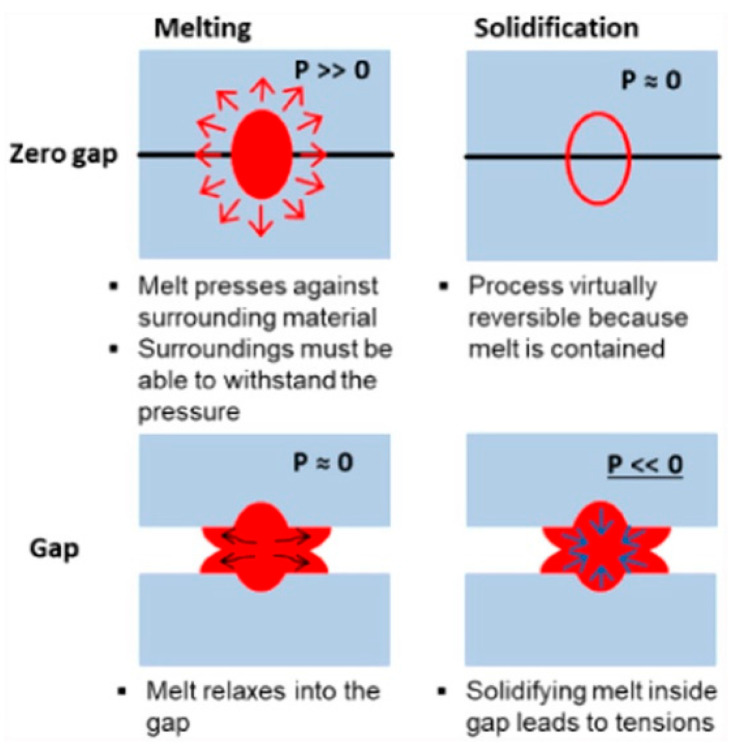
Schematic comparison between zero-gap and gap welding [109].

However, when utilizing a USP laser to weld glass, it is frequently necessary to reduce the gap by mechanical pressure or optical contact [108]. All of the experiments mentioned above require visual contact between the materials to weld successfully. The fundamental reason is that during the USP laser welding process, the laser’s nonlinear absorption by the material causes only a small quantity of material at the focal point to melt after absorbing the laser energy. As the amount of molten glass is restricted, it is insufficient to fill any leftover gaps. Figure 21 [109] depicts a schematic comparison of zero-gap welding versus gap welding, demonstrating that during the heating process—for example, during the heat-up—when the laser is absorbed by the material, the melt pool forms and thermal expansion occurs. In this zero-gap situation, enormous compressive stresses will result, causing the plates to separate and become damaged.

Previous studies have emphasized the importance of gap control: due to the limited volume of the molten zone, optical contact or mechanical clamping is usually required. However, subsequent experiments have shown that by optimizing the laser wavelength, focal position, and energy input, continuous welds can be achieved even across micron-scale gaps (approximately 3–4 μm).

Wu [110] used dual-pulse ultrashort lasers for glass welding. Their testing results showed that, as compared to traditional single-pulse laser welding, the dual-pulse technique increased the welding efficiency by about 22%.

**Figure 22:**
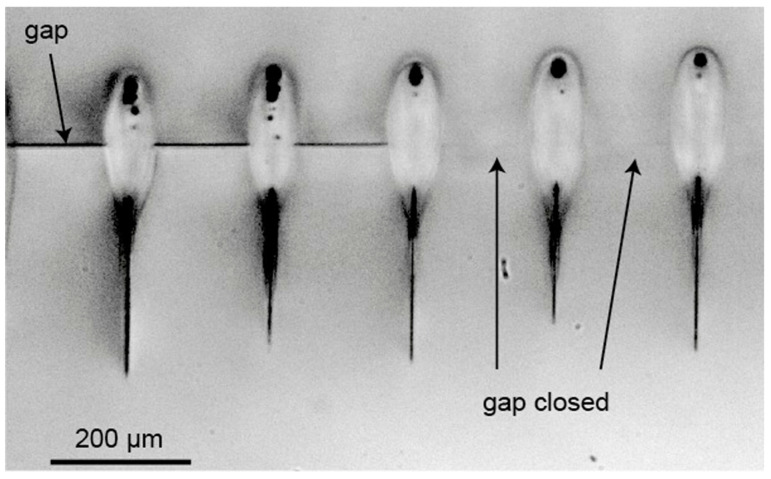
Side view on laser-welded samples [111].

Richter [111] used a femtosecond laser with a wavelength of 1030 nm and a pulse duration of 500 fs to weld quartz glass with a gap of 3~4 μm at a welding speed of 10 mm/s. As shown in Figure 22, after each scan, the focus position was moved upwards (*z*-axis) after each line. The gap had a size of approximately 4 µm. It can be observed that the gap on the right, which was initially 4 μm wide, has been closed by the weld seam, demonstrating the feasibility of using USP laser welding to reduce the gap. Miao [112] employed a green femtosecond laser with a wavelength of 515 nm, pulse width of 800 fs, and a maximum power of 75 W, together with a long-focus scanning mirror with a focal length of 255 mm, to fuse two pieces of 0.5 mm thick borosilicate glass with a gap of roughly 3 μm. They explored how the focal position, pulse energy, and scanning rate affected weld seam development.

**Figure 23:**
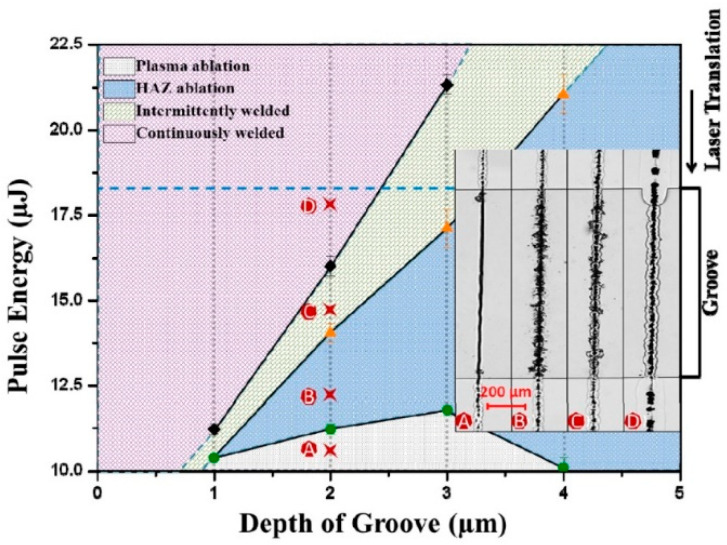
Parameter map illustrating the classification of weld results with varying pulse energy Q_0_ and gap distance G in fused silica. The inset shows four different welding patterns (f = 400 kHz, λ = 1030 nm, τp = 5.9 ps) (with courtesy of J. Chen et al.) [113].

Chen [113] demonstrated that picosecond laser welding can bridge micron-scale gaps without cracking between two pieces of glass with a 3 µm gap. It is demonstrated that the maximum gap that can be welded is not considerably impacted by the welding speeds, but is strongly influenced by the laser power and focus position relative to the interface between the materials. They demonstrated that the overlap welding of the glass plates with a gap can be divided into four categories: (A) plasma ablation, (B) HAZ (heat-affected zone) ablation, (C) intermittently welded, and (D) continuously welded (successful welding), as shown in the inset of Figure 23. The result depicts the weld bead obtained at different values of gap G (depth of groove) and the pulse energy Q_0_ at f = 400 kHz with a fixed laser focus location in fused silica, demonstrating that the pulse energy Q_0_ required for continuous welding (D) grows linearly with the gap.

Collectively, these findings confirm the high precision and industrial potential of ultrafast laser welding, while highlighting challenges in the energy deposition, heat accumulation, and gap tolerance, suggesting the need for further optimization and multiphysics-assisted strategies.

Nonlinear absorption rate analysis can be used to investigate the effects of laser pulse energy and the pulse repetition rate on USP lasers [114]. The proper control of thermal accumulation, the construction of a dual-structure molten pool, and the application of dual-pulse techniques can substantially enhance the welding efficiency and joint integrity. Moreover, adjusting the laser power and focal position during the welding process is critical to achieving optimal results. Once these issues are addressed, USP lasers can enable the crack-free welding of dissimilar glass-to-glass materials with significant differences in thermal properties such as CTE, thermal conductivity, and melting temperature [71,113].

Compared with traditional connection technologies, laser welding offers the following advantages:

1. Non-contact processing, where the heat source is unlikely to harm circuit components [115];
2. Enables localized connections with a low environmental impact [74];
3. Provides strong connections and excellent welding efficiency [116].

However, when considering practical industrial applications, increasing the processing throughput and achieving the automation of the welding process is a critical task. Although laser welding technology has significant advantages in its precision and weld quality, it remains in the experimental stage and has not been widely promoted due to its slower welding speed, high equipment costs, and low energy efficiency.

To provide a comprehensive comparison of current glass-joining techniques, Table 1 presents a structured summary of five representative methods—glass brazing, ultrasonic welding, ultrasonic-assisted brazing, anodic bonding, and laser welding. For each technique, the table highlights its core advantages, inherent limitations, typical application scenarios, and current status of industrial adoption. This comparative overview not only facilitates a clearer understanding of the fundamental differences among these joining approaches, but also serves as a practical reference for selecting suitable methods based on specific material requirements, design constraints, and performance expectations.

## 3. Future Trends in Glass Welding

Brazing, ultrasonic welding, anodic bonding, and laser welding all have unique benefits and play essential roles in a variety of applications. Brazing is commonly employed in large-scale production and the connecting of complex glass components due to its high joint strength and stability. Ultrasonic welding has slowly gained popularity in the glass manufacturing business due to its great efficiency and low energy consumption. Anodic bonding allows for the effective connection of miniature devices. The laser welding of nonmetallic materials marks a new frontier in laser applications. In particular, the connecting of transparent materials has long been recognized as a significant production challenge. With its high precision and localized heating capacity, the laser welding of glass has considerable application potential in the fabrication of high-performance optical components and micro-devices. The future development of glass welding technology is moving in several interrelated directions. For example, ultrasonic-assisted brazing and femtosecond laser welding can enhance the direct bonding between optical materials, resulting in joints with significantly higher shear strength. The combination of USP laser welding and direct bonding offers an innovative joining method that leverages the advantages of physical processes. In the future, further research can be conducted in the following areas to promote the broader application of glass technology.

Research on the interfacial compatibility mechanisms of different types of glass materials—such as silicate glass, borosilicate glass, and fused silica—under various energy fields (laser, electric field, and ultrasound) is essential for predicting the bonding performance and guiding the selection of suitable welding processes.

Based on the characteristics of CO_2_ laser welding, femtosecond laser welding, anodic bonding, and ultrasonic-assisted welding, the development of a multi-physics coupled simulation and experimental validation methods—capable of accurately predicting molten pool temperature fields, stress fields, defect formation mechanisms, thermal cycling, and crack propagation—will enable the optimization of multi-process parameter combinations to improve the weld quality and efficiency.

The development of advanced residual stress control and structural design strategies, including dual-structure configuration, intermediate layer introduction, and interfacial chemical modification, is critical for reducing crack risk and enhancing the long-term reliability of welded joints.

The integration of real-time monitoring technologies (e.g., infrared thermography and laser interferometry) with adaptive control algorithms will allow the dynamic adjustment of welding parameters during processing, thereby improving the process stability and joint uniformity. At the same time, laser welding should further increase the throughput and automation levels to meet the efficiency and cost requirements of industrial production, while promoting the development and application of portable, compact, and cost-effective welding systems in industry.

## Figures and Tables

**Table 1:** Comparative analysis of glass-bonding techniques.

Method	Advantages	Limitations	Typical Applications	Industrial Status
**Brazing of Glass**	High strength; good hermeticity	Residual stress from thermal mismatch; sensitive to glass composition	Micro-mechanical devices; vacuum equipment	Widely used in aerospace and optical assemblies
**Ultrasonic Welding**	Small heat-affected zone	Limited strength; requires smooth surfaces; rarely used directly on glass	-	-
**Ultrasonic-assisted Brazing**	Minimizes thermal damage; suitable for heat-sensitive glass	Risk of porosity at interface; oxidation if prolonged	Microfluidic devices; MEMS components	Experimental, emerging in precision applications
**Anodic Bonding**	High quality; exhibits good long-term stability	Requires smooth surface and matching CTE	MEMS sensors; microelectronic packaging	Widely adopted in MEMS and microsensors
**Laser Welding**	High precision; non-contact	High cost; needs good optical contact	Optical devices; MEMS sensors	Partially industrialized, mainly in optics

Data compiled from multiple sources [28,69,70,72,73,74,107,113,115,116] and representative of recent advances in MEMS and optical packaging.

## Data Availability

No new data were created or analyzed in this study. Data sharing is not applicable.
