# Correction: Yan et al. Advances of Welding Technology of Glass for Electrical Applications. *Materials* 2025, *18*, 4096

**DOI:** 10.3390/ma19091786

**Published:** 2026-04-28

**Authors:** Dejun Yan, Lili Ma, Jiaqi Lu, Dasen Wang, Xiaopeng Li

**Affiliations:** 1 Guangdong Provincial Key Laboratory of Advanced Welding Technology for Ships, Foshan University, Foshan 528011, China; lys@njust.edu.cn; 2 College of Materials Science and Technology, Nanjing University of Science and Technology, Nanjing 210014, China; malili0512@126.com (L.M.); 124116023919@njust.edu.cn (J.L.); 3 The Ningbo Branch of Ordnance Science Institute of China, Ningbo 315103, China; malili0512@163.com

In the original publication [1], there was an error regarding the affiliation for Dejun Yan. The affiliation has been updated to the following: Guangdong Provincial Key Laboratory of Advanced Welding Technology for Ships, Foshan University, Foshan 528011, China.

With this correction, the Conflicts of Interest statement has been adjusted accordingly. The original statement read “Author Dejun Yan was employed by the company China State Shipbuilding Corporation Huangpu Wenchong Shipbuilding Company Limited. The remaining authors declare that the research was conducted in the absence of any commercial or financial relationships that could be construed as a potential conflict of interest.” It has been changed to “The authors declare no conflicts of interest”.

The authors state that the scientific conclusions are unaffected. This correction was approved by the Academic Editor. The original publication has also been updated.
